# Micronutrients in Multiple Pregnancies—The Knowns and Unknowns: A Systematic Review

**DOI:** 10.3390/nu13020386

**Published:** 2021-01-27

**Authors:** Magdalena Zgliczynska, Katarzyna Kosinska-Kaczynska

**Affiliations:** 2nd Department of Obstetrics and Gynecology, Centre of Postgraduate Medical Education, 01-809 Warsaw, Poland; katarzyna.kosinska-kaczynska@cmkp.edu.pl

**Keywords:** twin pregnancy, multiple pregnancy, nutrition, micronutrients, supplementation

## Abstract

Maternal diet and nutritional status are of key importance with regard to the short- and long-term health outcomes of both the mother and the fetus. Multiple pregnancies are a special phenomenon in the context of nutrition. The presence of more than one fetus may lead to increased metabolic requirements and a faster depletion of maternal macro- and micro- nutrient reserves than in a singleton pregnancy. The aim of this systematic review was to gather available knowledge on the supply and needs of mothers with multiple pregnancies in terms of micronutrients and the epidemiology of deficiencies in that population. It was constructed in accordance with Preferred Reporting Items for Systematic Reviews and Meta-Analyses Statement (PRISMA). The authors conducted a systematic literature search with the use of three databases: PubMed/MEDLINE, Scopus and Embase. The last search was run on the 18 October 2020 and identified 1379 articles. Finally, 12 articles and 1 series of publications met the inclusion criteria. Based on the retrieved studies, it may be concluded that women with multiple pregnancies might be at risk of vitamin D and iron deficiencies. With regard to other microelements, the evidence is either inconsistent, scarce or absent. Further in-depth prospective and population studies are necessary to determine if nutritional recommendations addressed to pregnant women require adjustments in cases of multiple gestations.

## 1. Introduction

There is growing evidence that the nutritional status of pregnant women has a huge impact on the future health of the offspring [1,2,3,4]. Adequate maternal nutrient supply and supplementation in deficient populations may reduce the risk of numerous pregnancy complications, including premature birth, fetal growth disorders, pre-eclampsia and many others [5,6,7]. The well proven and globally recognized role of folic acid is worth mentioning in the prevention of neural tube defects in the offspring [8]. Moreover, increasing attention has recently been paid to the impact of fetal and early nutrition on the risk of noncommunicable diseases, such as cardiovascular diseases, diabetes, obesity or even neurocognitive impairments later in life [9]. For almost two decades, scientists around the world have been studying the so-called programming phenomenon related to the onset of such diseases [1,9,10,11,12].

Multiple pregnancies constitute a special challenge in terms of nutrition. The presence of more than one fetus contributes to a higher metabolic demand, thus increasing the risk of nutritional deficiencies [13,14,15]. Moreover, higher levels of chorionic gonadotropin observed in multiple pregnancies predispose to hyperemesis gravidarum, which may be another cause of maternal malnutrition [16,17]. Furthermore, pregnancies with an initially increased risk of numerous complications, including fetal growth disorders, pre-eclampsia, gestational diabetes or preterm labor may also constitute a contributing factor [18,19,20]. Therefore, it seems that the supply of nutrients should be properly tailored to the increased needs of a woman to effectively combat all potentially modifiable risk factors. Conversely, an excessive supply, which may be more common in developed countries, should be avoided. Inordinate supplementation, resulting from the simultaneous use of several multivitamin preparations, may also have a negative impact on the developing pregnancy, e.g., an increased risk of congenital defects in case of the excessive use of vitamin A [21]. Furthermore, it has recently been reported by numerous authors that unnecessary iron supplementation may contribute to negative pregnancy outcomes by increasing the risk of pregnancy-induced hypertension, pre-eclampsia or gestational diabetes mellitus [22,23,24,25,26].

Over the years, scientific organizations have attempted to create guidelines on this topic. For example, the National Institute for Health and Care Excellence (NICE) guidelines encouraged giving the same advice about diet, lifestyle and nutritional supplements to women with a twin or triplet pregnancies and those with singleton pregnancies [27]. However, at the same time, it was emphasized that anemia occurred more frequently in the former groups, so it was also recommended that additional blood tests be performed in order to rule out iron or folic acid deficiency, and that a dietitian be consulted during early pregnancy [27]. In 2006, the National Academy of Medicine (formerly the Institute of Medicine) issued the Consensus Study Report and suggested that women carrying more than one fetus may require the increased Recommended Dietary Allowances of folate, niacin, riboflavin and thiamin [28]. Another proposal of nutritional recommendations concerning the total daily intake of iron, calcium, vitamin D, magnesium, zinc, folic acid, vitamins C and E, broken down by trimesters, was presented in the Goodnight and Newman review for the Society of Maternal-Fetal Medicine (SMFM) [29]. Moreover, various specialized centers dedicated to such patients also attempted to create their own local guidelines [30,31,32]. Valuable and extensive research on this topic was conducted by Barbara Luke and her team [13]. In a publication from 2003, they described a specialized program focused on nutritional education and interventions in women pregnant with twins. Its implementation contributed to a significant improvement in the study group outcomes [33,34]. 

Nevertheless, the evidence is still scarce. The authors of the Cochrane Systematic Review conducted in 2015 did not find a single randomized or quasirandomized trial investigating whether specialized diets or nutritional advice for women with multiple pregnancies might improve their outcomes [35]. Moreover, in 2020, the SMFM issued a Special Statement on the state of the science on multifetal gestations, and emphasized that the potential benefits of focused nutritional intervention or micronutrients in multiple pregnancies still remained an unanswered question [36]. Therefore, the aim of this systematic review is to gather all the available knowledge on the supply and needs of mothers with multiple pregnancies with regard to micronutrients and the epidemiology of deficiencies in this population.

## 2. Materials and Methods

The review was developed in accordance with the guidelines presented in Preferred Reporting Items for Systematic Reviews and Meta-Analyses Statement (PRISMA) [37]. In order to retrieve manuscripts on the above-described topic, a systematic literature review was carried out with the use of three databases: PubMed/MEDLINE, Scopus and Embase. We did not stablish any time criterion. All articles found in the used databases were taken into account regardless of the publication date. The last search was run on the 18 October 2020. The detailed search strategy is presented in Table 1. 

The authors identified 1379 articles. With the use of the automatic search function in EndNote X9 (Clarivate Analytics, London, UK), 331 duplicates were discarded while 89 more were manually identified. The titles and abstracts of the remaining 959 manuscripts were screened by both study authors (M.Z., K.K.-K.) according to the inclusion and exclusion criteria (Table 2). 

**Figure 1:**
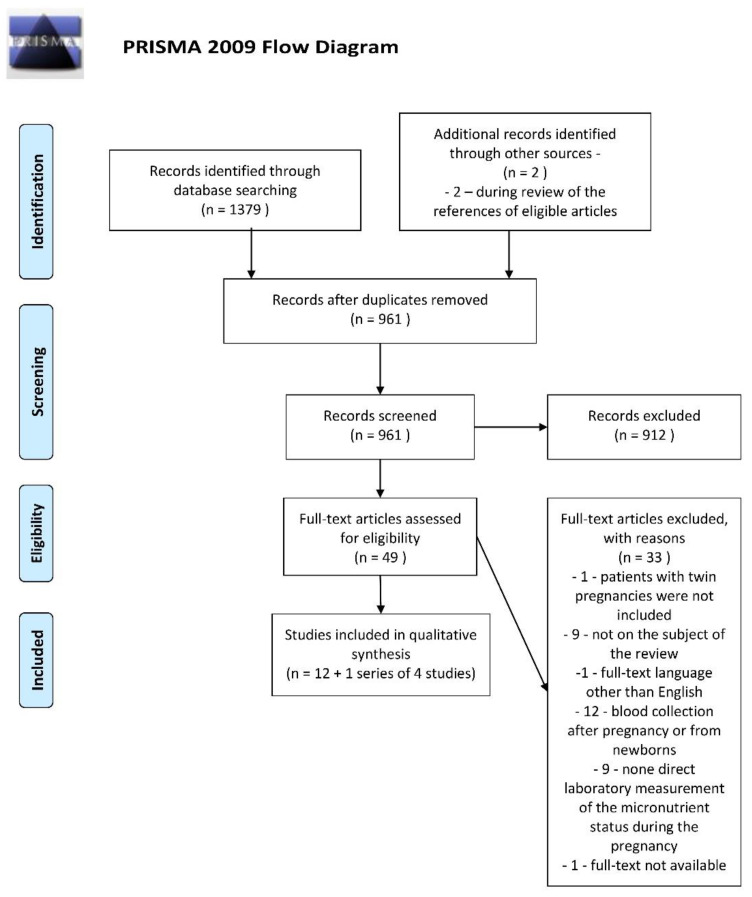
PRISMA Flow Diagram. From: Moher D, Liberati A, Tetzlaff J, Altman DG, The PRISMA Group (2009). Preferred Re-porting Items for Systematic Reviews and Meta-Analyses: The PRISMA Statement. PLoS Med 6(7): e1000097. doi: 10.1371/journal.pmed.1000097. For more information, visit: www.prisma-statement.org.

The references of the retrieved studies were also reviewed to identify additional articles. In this way, two more relevant papers were found [38,39]. Eligibility assessments were performed independently by both authors as the next step. Any disagreements in this regard were resolved through discussions between the authors. The details of the selection process are presented in a customized PRISMA Flow Diagram (Figure 1).

A customized data extraction sheet was subsequently used for the collection of the following information: authors, year and country of publication, the type of study, the main aim, population demographics, inclusion and exclusion criteria, diagnostic tools used and the results. One of the review authors extracted the above-mentioned data from eligible studies while the second author double-checked their correctness. 

The risk of bias was assessed using the AXIS tool (Supplementary Materials, Table S1) [40]. Additionally, other potential sources of bias, not included in the scale, were described in the “Risk of bias assessment” subsection in the Discussion section.

Attempts were made to contact the authors of five studies and one journal in order to obtain additional information [39,41,42,43,44,45,46]. Despite approaching the journal and authors of one potentially eligible article, we failed to access its full-text version [39]. Nwosu et al. were asked about the time of maternal vitamin D status measurements. The tests turned out to be performed after delivery, so the study had to be excluded from the review [41]. Delaney et al. clarified their data on serum ferritin levels in multiples [42]. We also contacted Nakayama et al. to obtain exact numerical data concerning serum calcium and serum phosphate in twin pregnancies presented in Figures 1A and 2A in their manuscript, as well as De la Calle et al., Bajoria et al. and Blarduni et al. about the mean maternal and/or gestational age in multiple pregnancies analyzed in their study. However, until publication, we have not received a reply or the data sought [43,44,45,46]. 

## 3. Results

### 3.1. Characteristics of the Retrieved Studies

The systematic literature search retrieved 12 articles and 1 series of publications (four articles) [42,43,44,45,46,47,48,49,50,51,52,53,54,55,56,57]. They included 830 participants from eight countries, predominantly with twin pregnancies (96%). Only 2.7% were mothers carrying three or more fetuses, whereas 1.3% were pregnancies of unknown type. With the exception of the papers by Ru et al. and Nakayama et al., patients were assessed at one time point, most often in the third trimester of pregnancy. The articles simultaneously assessed the supply of up to three micronutrients. Their main characteristics are collected in Table 3.

### 3.2. Vitamin D, Calcium and Phosphorus

Vitamin D, calcium and phosphorus were the most frequently studied microelements. Four studies showed lower levels of total 25-hydroxyvitamin D (25(OH)D), one did not reveal statistically significant differences between multiple and singleton pregnancies, and one showed higher concentrations in women expecting multiples. The results obtained for calcium and phosphorus were inconsistent. The main results of the studies are summarized in Table 4.

### 3.3. Iron

Several studies on iron status in multiple gestations have been published [42,45,52,56]. Ru et al. examined iron status in women with multiple pregnancies at two time points, i.e., during pregnancy and at delivery [52,53,54,55]. The majority of women received iron supplementation during pregnancy at various doses (82%; 27–90 mg/d). The authors proved that the depletion of iron stores (serum ferritin < 12 µg/L) was highly prevalent in this group (37% of women who provided samples during pregnancy and 26.2% at delivery). Women who had undergone assisted reproductive technologies (ARTs) had significantly higher mean serum ferritin at delivery than those from non-ART group: 32.4 (95%CI 23.0–45.5) versus 16.4 µg/L (95%CI 12.9–20.8). Iron deficiency anemia, defined as hemoglobin <11.0 g/dL in the first and third trimesters and hemoglobin concentrations of 10.5 g/dL in the second trimester, with the coexistence of deviations in iron status indicators was diagnosed in 15.1% of women during pregnancy and in 18% at delivery. Women with intragestational serum ferritin levels <12 µg/L and women with intragestational erythropoietin >75th percentile had a two-fold and three-fold higher risk, respectively, of anemia at delivery. The authors suggested that the abovementioned tests might become promising predictors [52]. A possible advantage of erythropoietin as a marker was related to the fact that it was not associated with either IL-6 or CRP levels, which made its levels independent of any underlying inflammatory processes. In a subsequent study, the authors showed that neonates born from multiple pregnancies were at a higher risk of anemia than reported for singletons. This was especially pronounced in smokers and mothers who were obese during pregnancy [53]. Analyses of determinants and variances in neonatal iron status were found in subsequent papers [54,55].

In a study group presented by Shinar et al., 67.3% of 446 women had hemoglobin values lower than 10.5 g/dL in the second trimester of twin pregnancies. The mean ferritin concentration was 16.0 µg/L (95% CI 11.6–17.5) and 42% of women had positive anemia workup results, including 34% with iron deficiency anemia (defined in the study as hemoglobin below 105 g/L with the coexistence of ferritin <15 µg/L). The hemoglobin value that was the best predictor of positive anemia workup results was 9.7 g/dL (sensitivity 80%, specificity 70%; AUC 0.809 ± 0.260) [56]. In a study by Delaney et al., one out of three women with multiple pregnancies for whom data on serum ferritin were available had a level below 12 μg/L [42]. Bajoria et al. showed that the mean maternal ferritin concentrations in TTTS and non-TTTS twin pregnancies were similar, with the mean close to the lower limit of the normal range, i.e., 30 and 29 µg/L, respectively [45].

### 3.4. Folic Acid and Vitamin B12

The authors of four studies investigated folic acid or/and vitamin B12 status in multiple pregnancies, including two studies conducted almost 60 years ago [42,47,48,52]. Ball and Giles analyzed fourteen patients with twin pregnancies. Two of them had megaloblastic anemia with the folic acid levels of 1.1 and 2.0 ng/mL. However, the second one was examined three days after delivery. In the remaining 12 patients, serum folic acid levels ranged from 1.1 to 5.3 ng/mL (mean: 2.6 ng/mL). This was significantly lower than the expected mean of 3.4 ng/mL calculated based on the results of singleton pregnant women at the same period of gestation. Nevertheless, those groups did not differ significantly in terms of vitamin B12 concentrations [47]. Scott and Sommerville used the Figlu test (histidine-loading test) to detect folic acid deficiencies. A total of 34 Figlu tests were carried out in 33 patients. The results indicated that 77% of the tests were positive, and 78% of them showed a good hematological correlation [48]. One initially negative test became positive six weeks later. Conversely, the authors of two contemporary studies, Ru et al. and Delaney et al., did not find features of folic acid and vitamin B12 deficiencies at the time of delivery in women with multiple pregnancies [42,52].

### 3.5. Other Studied Micronutrients

Only one eligible article identified other micronutrients [57]. Jantsch et al., who studied oxidative stress in twin pregnancies, proved that the level of vitamin C was significantly lower in twin than in singleton pregnancies. This was found to be accompanied by higher levels of thiobarbituric acid reactive substances, which are used for the detection of lipid peroxidation and the assessment of the intensification of oxidative stress phenomena [57].

### 3.6. Risk of Bias Assessment 

The risk of bias in the included studies was assessed using the AXIS tool [40]. The details of the evaluation of each paper are presented in Table S1 (See Supplementary Materials, Table S1). Overall, the most prominent potential source of bias visible in this group of articles resulted from the fact that a considerable proportion of the included studies provided no explanation concerning the size of the study group and the power of the tests performed (11 out of 13 studies). Moreover, the majority of authors did not characterize nonrespondents, reveal the response rate (9 out of 13) or discuss the limitations of their research (8 out of 13). Furthermore, almost half of them did not disclose potential conflicts of interest. However, these factors were mainly related to articles published over 10 years ago (6 out of 13). Other relevant study limitations not included in the scale are outlined in Section 4.3. Strengths and limitations of the study.

## 4. Discussion

### 4.1. Main Discussion

Appropriate diagnosis and prevention of micronutrient deficiencies in the population of women with multiple pregnancies is crucial, especially given the increasing frequency of twin pregnancies with the greater use of ARTs and late motherhood [58]. The majority of articles included in the review proved a high risk of micronutrient deficiencies in women with multiple gestations; most of them considered iron and vitamin D status, but only a handful of nonrandomized studies were included for each micronutrient. 

Most studies on vitamin D included in the review showed significantly lower levels of total 25(OH)D in multiple gestations in comparison with singletons. Only one study, by Okah et al., revealed that the serum concentrations of 25(OH)D were significantly higher in multiple than in singleton gestations and in other populations of mothers of twins studied by other authors [50]. The authors also tested the concentrations of other metabolites and, apparently, patients with twin pregnancies were also characterized by lower concentrations of 1.25-dihydroxyvitamin D (1.25(OH)_2_D). A similar finding was reported by Nakayama et al. in the cross-sectional part of their study, whereas Reddy et al. found no differences between singletons and multiples in terms of 1.25(OH)_2_D levels. Seemingly, the analysis of the individual metabolites of vitamin D along with the entire calcium-phosphate economy and bone-turnover markers would be advisable for a better understanding of this issue. Notably, the results of the retrieved studies indicated that when interpreting laboratory findings in multiples, it was crucial to consider corrected calcium, because women with multiple pregnancies seemed to have significantly lower albumin concentrations, i.e., often below the reference values for the general population [49,51].

All included studies clearly indicated that iron deficiency was common in the study group. However, an interesting association was reported in one study. Ru et al. discovered that women after ARTs had better iron supplies than the corresponding spontaneous pregnancy group [52]. This is a premise that draws attention to the diversity of such populations. First of all, ART pregnancies are always strictly planned, often after many years of trying to conceive. Moreover, they are at a higher risk of both maternal and neonatal complications [59]. This is definitely a topic for further exploration, including checking for unintended side effects of prolonged supplementation such as micronutrient overload.

The most recent of the retrieved articles, a study by Jantsch et al., was the only one that focused on a problem other than deficiency anemia or calcium and phosphate metabolism. Jantsch et al. associated lowered levels of vitamin C in twin pregnancies with greater consumption related to the increased production of free radicals in this type of pregnancy [57]. Similar reports were previously only available for animal models [60]. These findings may provide valuable clues, for example, for further research focused on counteracting pre-eclampsia or intrauterine growth disorders related to the phenomenon of oxidative stress. This seems especially important for women with multiple pregnancies, as this population is significantly more often affected.

Nevertheless, the main conclusion to be drawn from this study is the paucity of knowledge on the subject. Therefore, the indirect evidence of deficiencies in mothers with multiple pregnancies is also worth discussing. In an interesting literature review Miller et al. showed the overrepresentation of multiple pregnancies in correlation with young infants with metabolic bone disorders, including rickets [61]. However, the authors emphasized that the finding might have a multifactorial etiology in twins, with calcium or vitamin D deficiency being only one of the potential causes [61]. With regard to other microelements, a study was conducted in a population living in rural South India by Katz et al. They showed over a three-fold risk of maternal night blindness in a group of women with multiple pregnancies. The levels of vitamin A were not directly measured in the study. However, the association of night blindness during pregnancy with poor dietary vitamin A intake is well established [62]. Zhou et al. studied the correlation between nutrition during pregnancy in terms of vitamin A, vitamin D, calcium, iron, magnesium and zinc and the risk of astigmatism in twin neonates. They stated that 20% of mothers had serum nutrient deficiencies [63]. Nevertheless, more detailed data were not presented in the study. 

With regard to the iron status in women with multiple gestations, valuable data were also found, but could not be included in this systematic review. Hediger and Luke, who presented a part of their results at the American Public Health Association Meeting in 1999, showed that the mean third trimester ferritin levels were low, i.e., 12 ng/mL, or the cut-off for the diagnosis of iron deficiency, in the mothers of twins. Interestingly, ferritin levels were lower in women with higher maternal weight gain before 24 gestational weeks and with a greater birth weight of children. The authors concluded that the low levels of ferritin in the third trimester, even those indicating iron deficiency, might actually be a good clinical indicator of fetal growth, as iron stores seemed to be intensively exploited for the support of multiple fetus growth [34,64]. In addition, studies comparing hemoglobin levels in singleton and twin pregnancies were also published. For example, Blickstein et al. confirmed lowered levels in women expecting twins [65]. Spellacy et al. reported that anemia occurred 2.4 times more commonly in this group [66]. It is worth mentioning the results of one of the included studies published by Shinar et al. The authors showed that a hemoglobin cutoff of 9.7 g/dL was the best predictor of deficiency anemia in a group of women with twin pregnancies. Therefore, it might be necessary to reevaluate hemoglobin reference ranges for multiples, since, in this case, the phenomenon of hemodilution may have a greater impact on its levels. 

### 4.2. Implications and Future Research Directions

The University of Michigan Multiples Program focused on the reduction of maternal and neonatal complications by the implementation of complex actions aimed at optimizing nutritional status through close monitoring, appropriate education and influence on as many modifiable factors as possible [33]. Pregnant women included in the program more commonly met weight gain goals, whereas neonates were less frequently preterm, had higher birth weights and required fewer medical interventions. The positive effect of this intervention was related to the fact that children were at a lower risk of hospitalization over the first three years of life and were less likely to have any delayed neurologic development [33,34]. Furthermore, the savings at birth were estimated to be $14,023 per infant for Program twins. A similar study (the Higgins Nutrition Intervention Program) also showed promising results [67]. In conclusion, the results of such studies are promising and should provide motivation to prioritize further research in this area. The huge number of “unknowns” in the title which remain after analyzing the literature also indicate the urgent need for further research. First, research on the epidemiology of deficiencies in women pregnant with multiples on larger groups of patients and on a wider range of vitamins and micronutrients are essential to identify which deficiencies this population is particularly vulnerable to. Next, longitudinal prospective studies are necessary to assess the nutritional status of individual microelements in subsequent trimesters of pregnancy, also considering other factors, e.g., ART history, chorionicity, the cooccurrence of pregnancy complications and diet. The last item seems to be especially noteworthy, since balanced diet may help to maximize the absorption of beneficial nutrients. For example, several flavonoids have been recently shown to decrease the bioavailability of some micronutrients, mostly iron, due to the formation of nonabsorbable chelates during digestion [68]. Finally, if a higher risk of deficiencies is confirmed, randomized controlled trials and large population studies could help to determine appropriate supplementation guidelines. In such cases, it would also be advisable to screen neonates born from multiple pregnancies for potential deficiencies as well as patients with a history of multiple pregnancies for long-term complications due to nutritional deficiencies.

### 4.3. Strengths and Limitations of the Study

The first limitation of the study is the above mentioned significant risk of bias in the included articles. It needs to be mentioned that the eligible studies were mostly carried out in small groups. They were also neither randomized nor interventional studies. Another factor that may hinder the joint interpretation of study results is the variety of methods used. First of all, the studies were conducted on populations that differed in terms of factors presumably affecting the initial risk of nutritional deficiencies, e.g., race and ethnicity, maternal and gestational age, the number of fetuses, body mass index, financial status, a diet, the type of fertilization, local recommendations for supplementation in pregnant women. Moreover, the diversity of assays, machines, as well as implemented techniques, might also have led to biases. 

In addition to the quality of the included studies, another limitation of this review is the inability to perform quantitative analyses due to the paucity, but also the variety, of the collected data. Conversely, the noteworthy advantages of this systematic review are, first of all, the originality of the subject and the large number of papers reviewed by the authors.

## 5. Conclusions

Based on the available studies, it may be concluded that women with multiple pregnancies might be at a higher risk of vitamin D and iron deficiencies. For other microelements, the evidence is either inconsistent, scarce or absent. This topic requires further in-depth prospective and population studies. Nevertheless, the nutritional recommendations and schedule of tests aimed at the detection of key nutrient deficiencies in pregnant women probably require adjustments in cases of multiple gestations.

## Figures and Tables

**Table 1:** Databases and the search strategy.

Database	Search Strategy	Number of Results
PubMed/MEDLINE	(“pregnancy, multiple”(MeSH Terms) OR “twin pregnanc *”(Title/Abstract) OR “twin gestation *”(Title/Abstract) OR “twins”(Title/Abstract) OR “triplet *”(Title/Abstract) OR “quadruplet *”(Title/Abstract) OR “quintuplet *”(Title/Abstract) OR “multiple pregnanc *”(Title/Abstract) OR “multiples”(Title/Abstract) OR “multifetal *”(Title/Abstract)) AND (“Nutrients”(MeSH Terms) OR “nutrient *”(Title/Abstract) OR “Micronutrients”(MeSH Terms) OR “micronutrient *”(Title/Abstract) OR “Trace Elements”(MeSH Terms) OR “trace element *”(Title/Abstract) OR “Vitamins”(MeSH Terms) OR “vitamin *”(Title/Abstract))	542
Embase	(‘twin pregnanc *’:ab OR ‘twin gestation *’:ab OR twins:ab OR triplet *:ab OR quadruplet *:ab OR quintuplet *:ab OR ‘multiple pregnanc *’:ab OR ‘multiple gestation *’:ab OR multiples:ab OR ‘multifetal *’:ab) AND (nutrient *:ab OR micronutrient *:ab OR ‘trace element *’:ab OR vitamin *:ab) AND [embase]/lim	612
Scopus	(TITLE-ABS-KEY ((“twin pregnanc *” OR “twin gestation *” OR twins: OR triplet * OR quadruplet *: ab OR quintuplet * OR “multiple pregnanc *” OR “multiple gestation *” OR multiples OR multifetal *)) AND TITLE-ABS-KEY ((nutrient * OR micronutrient * OR “trace element *” OR vitamin *)))	225
3 databases		1379

Asterisk (*) represents any group of characters, including no character

**Table 2:** Inclusion and exclusion criteria for retrieved studies.

Inclusion Criteria	Exclusion Criteria
All types of original articles including case reports, case series and scientific letters, concerning the nutritional status of selected microelements in women with multiple pregnancies	Reviews, editorials, conference papers or abstracts
Published in a peer-reviewed scientific journal	Published in a journal other than a peer-reviewed scientific one
At least one laboratory measurement of the micronutrient level in women during the multiple gestation or at birth with available direct results	Post-pregnancy measurements
	Presumption of nutritional status based only on such factors as symptoms, not confirmed with a laboratory examination
Full-text available in English	Language other than English; only abstract available in English
Unrestricted publication time	
Human studies	Animal studies

**Table 3:** The main characteristics of the retrieved studies.

Study	Year and Country of Publication	Micronutrient(s) of Interest	Main Aim	Number of Women and Type of Multiple Pregnancy Included in the Study Results	Mean * Maternal Age at Examination (Years)	Mean * Gestational Age at Examination (Gestational Weeks)
Ball & Giles [47]	1964 UK	Folic acid Vitamin B12	To analyze folic acid and vitamin B12 levels in pregnancy and their relation to megaloblastic anemia	13 ** twin	26.7 ** ± 6.3	33.3 ** ± 5.6
Scott & Sommerville [48]	1965 UK	Folic acid	To perform a practical evaluation of the Figlu test in pregnancy	33 twin	LoD	LoD
Reddy et al. [49]	1983 Canada	Vitamin D	To establish the relationship of vitamin D metabolites with each other and their dependency on pregnancy-related hormones	27 twin	27 ± 4.3	Range 37–41
Okah et al. [50]	1996 USA	Vitamin D Calcium Phosphorus	To check if maternal bone turnover and mineral stress are greater in multiple pregnancy than in singleton pregnancy	15 twin 2 triplet	29.2 ± 1.5	30.6 ± 0.9
Bajoria et al. [45]	2001 UK	Iron	To investigate fetal iron metabolism in MC twin pregnancies in relation to TTTS	22 twin	LoD	TTTS 23 (Range 19–31) Control 30 (Range 21–34)
Nakayama et al. [43]	2011 Japan	Vitamin D Calcium Phosphorus	To examine the influence of twin pregnancy on calcium metabolism by comparison to singleton pregnancy	C-S study: 131 twin L study: 11 twin	L study: 30.8 ±4.8	Time points: 10, 25, 30 and 36 weeks
de la Calle et al. [44]	2016 Spain	Vitamin D	To answer if women pregnant with twins have lower serum levels of vitamin D	97 twin	LoD	Range: 12–14
Goswami et al. [51]	2016 India	Vitamin D Calcium Phosphorus	To compare maternal and neonatal vitamin D status in twin versus singleton pregnancies	50 twin	27.4 ± 4.2	35.8 ± 1.9
Ru et al. [52] and related studies: [53,54,55]	2016 USA	Iron Folic acid Vitamin B12	To characterize longitudinal changes in iron status across the pregnancy in a cohort of healthy women with multiple gestations and identify the determinants of maternal iron deficiency and anemia	64 twin 18 triplet 1 quadruplet	30.3 ± 5.1	Samples obtained during pregnancy 24.4 ± 5.4 at delivery 35.3 ± 2.3
Shinar et al. [56]	2018 Israel	Iron	To assess the benefit of the hemoglobin cutoff of 10.5 g/dL as a trigger for anemia evaluation during the second trimester of pregnancy	300 twin	25.8 ± 5.0	17.0 ± 0.9
Blarduni et al. [46]	2019 Spain	Vitamin D	To measure the prevalence of hypovitaminosis D in mothers and newborns	11 type unknown	LoD	LoD
Delaney et al. [42]	2020 USA	Iron Folic Acid Vitamin B12	To evaluate maternal iron absorption and identify factors associated with iron partitioning between the maternal, neonatal, and placental compartments	4 twin 1 triplet	31.0 ± 2.3	33.8 ± 2.5
Jantsch et al. [57]	2020 Brazil	Vitamin C	To assess maternal oxidative stress in twin pregnancies	30 twin	28.0 (interquartile range 23–33)	31.5 ± 0.5

Abbreviations: *—unless otherwise stated; **—the authors of the study made calculations in 14 women, but one of them had delivered 3 days before the laboratory examination, so we excluded her from the calculations in this table; ±—standard deviation; C-S—cross-sectional; L—longitudinal; LoD—lack of data; MC—monochorionic; TTTS—twin-to-twin transfusion syndrome; y.o.—years old; UK—United Kingdom; USA—United States of America.

**Table 4:** Main results of the studies on calcium, phosphorus and vitamin D status in multiple pregnancies.

Study	Data on Supplementation	Serum Calcium [Mean ± SD; mg/dL] *	Serum Phosphorus [Mean ± SD; mg/dL] *	Serum 25(OH)D [Mean ± SD; ng/mL] *		Comparison: Multiple vs. Singleton Pregnancies
Reddy et al. [49]	Women were advised to take 400 IU of vitamin D2	**2nd trimester**	**2nd trimester**	**2nd trimester**		- Serum calcium levels at delivery were significantly lower in multiple pregnancies (7.71 ± 0.45 vs. 8.6 ± 0.2 mg/dL) and correlated with serum albumin - Serum phosphorus and 25(OH)D levels were lower in multiple pregnancies (at delivery: 2.59 ± 0.24 vs. 3.2 ± 0.1 mg/dL; 11 ± 1.6 vs. 16.4 ± 2.8 ng/mL), but the authors did not provide data on the statistical significance
		8.96 ± 0.23	3.09 ± 0.12	15 ± 2.3		
		**3rd trimester**	**3rd trimester**	**3rd trimester**		
		8.75 ± 0.14	3.16 ± 0.09	14 ± 0.9		
		**At delivery**	**At delivery**	**At delivery**		
		7.71 ± 0.45	2.59 ± 0.24	11 ± 1.6		
Okah et al. [50]	Average daily dietary intake: Vitamin D: 1181 ± 246 IU Calcium: 1621 ± 346 mg	**3rd trimester**	**3rd trimester**	**3rd trimester**		- No significant differences were present in serum calcium and phosphorus levels between groups - Serum 25(OH)D levels were significantly higher in multiple gestations (61 ± 5 vs. 39 ± 2 ng/mL)
		9.1 ± 0.1	3.2 ± 0.2	61 ± 5		
Nakayama et al. [43]	Average daily dietary intake: Vitamin D: 296 ± 136 IU Calcium: 795 ± 287 mg Phosphate: 1022 ± 204 mg	See Figures 1B and 2B in the original manuscript ** [43]	See Figures 1B and 2B in the original manuscript [43]	**C-S study**	**L study**	- In the C-S study serum calcium and phosphorus levels were significantly **higher** in multiple pregnancies (See Figures 1B and 2B in the original manuscript [43]); in the L study no significant differences were present - 25(OH)D levels were lower in multiple pregnancies in the C-S and L studies (See Figures 1B and 2B in the original man-uscript [43]); in the L study the difference was significant only at 25 and 30 wks: 12.1 ± 4.5 vs. 26.3 ± 7.3; 13.8 ± 5.0 vs. 27.4 ± 10.7 ng/mL
				**10 weeks**	**10 weeks**	
				12.8 ± 5.1	14.6 ± 8.7	
				**25 weeks**	**25 weeks**	
				12.5 ± 4.3	12.1 ± 4.5	
				**30 weeks**	**30 weeks**	
				15.1 ± 5.5	13.8 ± 5.0	
				**36 weeks**	**36 weeks**	
				15.0 ± 6.6	14.2 ± 6.4	
de la Calle et al. [44]	All participants had taken multivitamin complex including 200 IU of vitamin D3 daily for at least 3 weeks before	NS	NS	**1st trimester**		- No significant differences were present in serum 25(OH)D levels between groups
				21.4 ± 7.8		
				90% < 30		
Goswami et al. [51]	Average daily dietary intake: Calcium: 1023 ± 275 mg No vitamin D supplementation was prescribed	**3rd trimester** 9.9 ± 0.66 **	**3rd trimester** 3.9 ± 0.76	**3rd trimester** 5.7 ± 4.2 90% < 12		- No significant differences were present in serum adjusted calcium levels and phosphorus between groups - Serum 25(OH)D levels were significantly lower in multiple pregnancies 5.7 ± 4.2 vs. 7.4 ± 4.9 ng/mL) - Maternal serum 25(OH)D showed a significant correlation with both cord blood serum 25(OH)D (r = 0.64 (1st twin); r = 0.65 (2nd twin))
Blarduni et al. [46]	LoD	NS	NS	92.3% < 20		- Multiple pregnancy was a risk factor of maternal hypovitaminosis D (<20 ng/mL) (OR: 6.29 (95%CI 1.41; 28.18))

Abbreviations: *—unless otherwise stated; **—corrected by serum albumin; 25(OH)D—total 25-hydroxyvitamin D; C-S—cross-sectional; L—longitudinal; LoD—lack of data, including no specific data for the multiple pregnancy population; NS—not studied; SD—standard deviation; weeks—gestational weeks.

## Supplementary Materials

The following are available online at https://www.mdpi.com/2072-6643/13/2/386/s1, Table S1. Risk of bias assessment.
