# Supplementary material for: Micronutrients in Multiple Pregnancies—The Knowns and Unknowns: A Systematic Review
Source: Nutrients. 2021 Jan 27;13(2):386. doi: 10.3390/nu13020386 (PMC7912422; doi:10.3390/nu13020386)
Supplement: Supplementary file 1 [file nutrients-13-00386-s001.pdf]

Table S1

Risk of bias assessment

Note: This is AXIS tool developed for a critical assessment of the quality of cross-sectional studies [1]

Possible answers: Yes / No / Do not know/comment

The assessment refers to the population of women with multiple pregnancies included in each study.

	Ball & Giles 1964	Scott & Sommerville	Reddy et al. 1983	Okah et al. 1996	Bajoria et al. 2001	Nakayama et al. 2011	de la Calle et al. 2016	Goswami et al. 2016	Ru et al. 2016	Shinar et al. 2018	Blarduni et al. 2019	Delaney et al. 2020	Jantsch et al. 2020
Introduction													
1. Were the aims/objectives of the study clear?	No	No	Yes	Yes	Yes	Yes	Yes	Yes	Yes	Yes	Yes	Yes	Yes
Methods													
2 Was the study design appropriate for the stated aim(s)?	Yes	Yes	Yes	Yes	Yes	Yes	Yes	Yes	Yes	Yes	Yes	Yes	Yes
3 Was the sample size justified?	No	No	No	Yes	No	No	No	No	Yes	No	No	No	No
4 Was the target/reference population clearly defined? (Is it clear who the research was about?)	Yes	Yes	Yes	Yes	Yes	Yes	Yes	Yes	Yes	Yes	Yes	Yes	Yes
5 Was the sample frame taken from an appropriate population base so that it closely represented the target/reference population under investigation?	Yes	Yes	Yes	Yes	Yes	Yes	Yes	Yes	Yes	Yes	Yes	Yes	Yes
6 Was the selection process likely to select subjects/participants that were representative of the target/reference population under investigation?	Yes	Yes	Yes	Yes	Yes	Yes	Yes	Yes	Yes	Yes	Yes	Yes	Yes
7 Were measures undertaken to address and categorize non-responders?	No	No	No	No	No	Yes	No	Yes	Yes	No	Yes	No	No

8 Were the risk factor and outcome variables measured appropriate to the aims of the study?	Yes	Yes	Yes	Yes	Yes	Yes	Yes	Yes	Yes	Yes	Yes	Yes	Yes
9 Were the risk factor and outcome variables measured correctly using instruments/ measurements that had been trialled, piloted or published previously?	Yes	Yes	Yes	Yes	Yes	Yes	Yes	Yes	Yes	Yes	Yes	Yes	Yes
10 Is it clear what was used to determined statistical significance and/or precision estimates? (e.g., p values, CIs)	Yes	No	Yes	Yes	Yes	Yes	Yes	Yes	Yes	Yes	Yes	Yes	Yes
11 Were the methods (including statistical methods) sufficiently described to enable them to be repeated?	Yes	No	Yes	Yes	Yes	Yes	No	Yes	Yes	Yes	Yes	Yes	Yes
Results													
12 Were the basic data adequately described?	No	No	Yes	Yes	Yes	Yes	Yes	Yes	Yes	Yes	Yes	Yes	Yes
13 Does the response rate raise concerns about non-response bias?	ND	ND	ND	ND	ND	No	ND	No	No	ND	No	ND	ND
14 If appropriate, was information about non-responders described?	No	No	No	No	No	Yes	No	Yes	Yes	No	Yes	No	No
15 Were the results internally consistent?	Yes	Yes	Yes	Yes	Yes	Yes	Yes	Yes	Yes	Yes	Yes	Yes	Yes
16 Were the results for the analyses described in the methods, presented?	Yes	ND	Yes	Yes	Yes	Yes	Yes	Yes	Yes	Yes	Yes	Yes	Yes
Discussion													
17 Were the authors' discussions and conclusions justified by the results?	Yes	Yes	Yes	Yes	Yes	Yes	Yes	Yes	Yes	Yes	Yes	Yes	Yes
18 Were the limitations of the study discussed?	No	No	No	No	No	Yes	No	No	Yes	Yes	No	Yes	No
Others													
19 Were there any funding sources or conflicts of interest that may affect the authors' interpretation of the results?	NDis	NDis	NDis	NDis	NDis	NDis	No	No	No	No	No	No	No

20 Was ethical approval or consent of participants attained?	NS	NS	NS	Yes	Yes	Yes	Yes	Yes	Yes	Yes	Yes	Yes	Yes

Abbreviations: ND – not described; NDis – not disclosed; NS – not stated

1. Downes, M.J.; Brennan, M.L.; Williams, H.C.; Dean, R.S. Development of a critical appraisal tool to assess the quality of cross-sectional studies (AXIS). *BMJ Open* **2016**, *6*, e011458, doi:10.1136/bmjopen-2016-011458.
